# Sex disparities in food consumption patterns, dietary diversity and determinants of self-reported body weight changes before and amid the COVID-19 pandemic in 10 Arab countries

**DOI:** 10.3389/fpubh.2022.1029219

**Published:** 2022-10-28

**Authors:** Reema Tayyem, Mohammed O. Ibrahim, Hussein Mortada, Majid AlKhalaf, Khlood Bookari, Haleama Al Sabbah, Radwan Qasrawi, Iman Kamel, Somaia Dashti, Sabika Allehdan, Hiba Bawadi, Mostafa Waly, Haya Abuhijleh, Fadwa Hammouh, Narmeen Al-Awwad, Nahla Al-Bayyari, Leila Cheikh Ismail, Diala Abu Al-Halawa, Manal Othman, The Regional CORONA COOKING Survey Group, Charlotte De Backer, Maha Hoteit

**Affiliations:** ^1^Department of Human Nutrition, College of Health Sciences, Qatar University (QU)-Health, Qatar University, Doha, Qatar; ^2^Department of Nutrition and Food Technology, Faculty of Agriculture, University of Jordan, Amman, Jordan; ^3^Department of Nutrition and Food Technology, Faculty of Agriculture, Mu'tah University, Karak, Jordan; ^4^Faculty of Science IV, Lebanese University, Beirut, Lebanon; ^5^National Nutrition Committee, Saudi Food and Drug Authority, Riyadh, Saudi Arabia; ^6^Department of Clinical Nutrition, Faculty of Applied Medical Sciences, Taibah University, Madinah, Saudi Arabia; ^7^Department of Health Sciences, College of Natural and Health Sciences, Zayed University, Dubai, United Arab Emirates; ^8^Department of Computer Science, Al-Quds University, Jerusalem, Palestine; ^9^Department of Computer Engineering, Istinye University, Istanbul, Turkey; ^10^National Research Centre, Cairo, Egypt; ^11^Public Authority for Applied Education and Training, Kuwait City, Kuwait; ^12^Department of Biology, College of Science, University of Bahrain, Zallaq, Bahrain; ^13^Food Science and Nutrition Department, College of Agricultural and Marine Sciences, Sultan Qaboos University, Muscat, Oman; ^14^Department of Nutrition and Dietetics, Health Sciences Faculty, American University of Madaba, Madaba, Jordan; ^15^Department of Clinical Nutrition and Dietetics, Faculty of Applied Health Sciences, Hashemite University, Zarqa, Jordan; ^16^Department of Nutrition and Food Technology, Faculty of Al-Huson University College, Al-Balqa Applied University, As-Salt, Jordan; ^17^Department of Clinical Nutrition and Dietetics, University of Sharjah, Sharjah, United Arab Emirates; ^18^Nuffield Department of Women's and Reproductive Health, University of Oxford, Oxford, United Kingdom; ^19^Department of Faculty of Medicine, Al Quds University, Jerusalem, Palestine; ^20^Faculty of Public Health, Lebanese University, Beirut, Lebanon; ^21^PHENOL Research Group (Public Health Nutrition Program Lebanon), Faculty of Public Health, Lebanese University, Beirut, Lebano; ^22^Lebanese University Nutrition Surveillance Center, Lebanese Food Drugs and Chemical Administrations, Lebanese University, Beirut, Lebanon; ^23^University Medical Center, Lebanese University, Beirut, Lebanon

**Keywords:** COVID-19 pandemic, dietary diversity, Arab countries, sex, body mass index, overweight

## Abstract

**Background:**

The COVID-19 pandemic along with its confinement period boosted lifestyle modifications and impacted women and men differently which exacerbated existing gender inequalities. The main objective of this paper is to assess the gender-based differentials in food consumption patterns, dietary diversity and the determinants favoring weight change before and amid the COVID-19 pandemic among Arab men and women from 10 Arab countries.

**Methods:**

A cross-sectional study was conducted based on a convenience sample of 12,447 households' family members (mean age: 33.2 ± 12.9; 50.1% females) and information from participants aged 18 years and above was collected about periods before and during the pandemic.

**Results:**

Findings showed that, during the COVID-19 period, the dietary diversity, declined by 1.9% among females compared to males (0.4%) (*p* < 0.001) and by 1.5% among overweight participants (*p* < 0.001) compared to their counterparts.

**Conclusions:**

To conclude, gender-sensitive strategies and policies to address weight gain and dietary diversity during emergent shocks and pandemics are urgently needed in the region.

## Introduction

The COVID-19 pandemic, declared by the World Health Organization (WHO) on March 11, 2020 caused a devastating blow to already-fragile gender-responsive policies and recovery plans among Arab countries (1, 2). According to solid evidence, women and men are experiencing the pandemic differently (2). For instance, many challenges were faced by women including food insecurity, unhealthy dietary patterns and malnutrition amid the unprecedented pandemic (3). The deteriorating food security situation amid the COVID-19 pandemic, suggests a greater impact on the diet diversity and daily nutrient intake in female households that was more reported than in male households (4). Moreover, the closure of school and daycare facilities during the pandemic resulted in a significant increase in childcare needs, which has adversely affected the social and health statuses of working mothers (5). Consequently, the pandemic caused considerable disruptions in women's daily routines, which may have an unanticipated impact on eating habits (6). For instance, women tend to eat more than men under stressful conditions, and men were less likely than women to feel guilty after an emotional eating episode (6). Other than that, women across many countries were found to modify their shopping habits more often when compared to men (3).

Individuals with overweight including women were also affected by the COVID-19 pandemic, especially during the first waves in which home confinement had negative repercussions on their dietary diversity, intake patterns, physical activity (7) and weight management (2, 8). Only four studies have investigated the effects of COVID-19 home isolation on weight change among men and women in Arab countries (9–12). Thus, based on the results obtained concerning the COVID-19 home isolation on weight status, food consumption patterns and dietary diversity, we decided to (1) assess the sex-based differentials in self-reported food consumption patterns, dietary diversity and self-reported body weight changes before and amid the COVID-19 pandemic; (2) examine the impact of poor dietary behaviors and low dietary diversity on the BMI; and (3) investigate the determinants favoring self-reported weight change among Arab men and women from 10 Arab countries.

## Materials and methods

### Study design and study instrument

The study instrument used was an online survey employed in a previous cross-sectional study that was initially launched in 38 different countries (13). Data from residents of ten Arab countries (Bahrain, Egypt, Jordan, Kuwait, Oman, Qatar, Saudi Arabia, United Arab Emirates, Lebanon, and Palestine) who took part in this survey were chosen for analysis in this study. The survey questions were available in Arabic and several other languages, giving respondents more options^1^. This was a collaborative work conducted in 38 countries under the project named: CORONACOOKING survey available on this link (https://www.uantwerpen.be/en/projects/food-media-society/corona-cooking-survey/).

The survey consisted of multiple blocks of questions and was open from April 17th to June 25th, 2020. Participants in this study were over the age of 18 and of both sexes who lived in 10 Arab countries during the COVID-19 crisis. To recruit participants, convenient sampling was used, and the survey was advertised on various social media platforms. The questionnaire was a validated online survey that was described previously (3, 14) (see text footnote 1). Full details of the methods used across all of the countries involved can be found in the original cross-sectional study described by De Backer et al. (13). The questionnaire consisted of a validated online survey to collect information related to different topics including sociodemographic and economic information, lockdown measures, cooking attitudes, shopping, food stock, and food frequency consumption in term of food portions per week [The question asked was: “how often did you eat the following (portions of) foods? Please indicate how often you consumed at least one portion of the following foods and drinks”] (13). Regarding questions related to cooking attitudes, shopping and food frequency consumption, respondents were asked to answer each question two times, indicating their behavior in both periods (before the COVID-19 pandemic and during the COVID-19 lockdown). The Food Consumption Score (FCS), a proxy indicator used for dietary diversity analysis, was calculated by taking the frequency of consumption of various food groups by a household in the 7 days preceding the survey. Details about the calculation formula of the FCS were described elsewhere (3). Anthropometric measures were self-reported by participants due to the safety restrictions imposed by the countries in this study. Body mass index (BMI) was calculated using a person's height and weight. Overweightness was reported when BMI was 25.0 kg/m^2^, while the healthy range was 18.5–24.9 kg/m^2^ (15). A binary logistic regression was calculated to look for factors that may influence the BMI among Arab men and women from 10 countries. The BMI (high vs. normal) was the dependent variable in this regression. A backward regression method was used, and factors with a *p*-value < 0.05 were considered significant. For each of the factors, the odds ratio (OR) and confidence interval were calculated.

### Ethical consideration

A consent form was obtained at the start of the study. Given that this study was observational with respect to confidentiality and no traceability of respondents, it was approved by the ethics committees at the University of Antwerp (SHW_46) (the project's lead country) and all other countries involved in the research.

### Statistical analysis

For categorical variables, respondent characteristics were presented as frequencies (percentages), whereas continuous variables were presented as mean standard deviation (SD). The results were assessed for all participants as well as for females and males separately. The Chi-square test was used to investigate differences in categorical variables between groups (sex). Mann-Whitney U test was used to compare differences between two independent groups. The independent *t*-test was used for continuous variables, and the Marginal Homogeneity test was used to distinguish between paired data (comparison before and during COVID-19) for males and females separately. Binary logistic regression was conducted to investigate the factors that may affect BMI change. In this regression, the BMI (High vs. normal) was the dependent variable. A backward approach was used and factors having a *p*-value < 0.05 were considered significant. The Odds Ratio (OR) and its confidence interval were also calculated for each of the factors. A *p*-value < 0.05 (typically ≤ 0.05) is considered statistically significant. IBM SPSS Statistics for Mac, Version 24, was used for statistical analysis.

## Results

### Sociodemographic and socioeconomic characteristics

Table 1 presents the sociodemographic and socioeconomic characteristics of the study participants. Overall, 12,447 family members from households in 10 Arab countries completed the survey and were used in the subsequent analysis. The study population had almost the same proportion of females and males, representing 50.1 and 49.9%, respectively. The mean age of the population was 33.2 years, of which 65.7% were adults (24–64 years old) and 28.6% were youth (19–24 years old). However, only a few participants were adolescents (18 years) or elderly, making up 3.9 and 1.8% of the total study population, respectively. Overall, 55% of the study population had a bachelor's degree, with 50.4% being males and 59.7% being females (*p*-value < 0.001). Also, 7.1% of the total study participants had doctorates, and the majority were males making 9.7% of the total percentage, vs. 4.6% of females. The Employment status changed during the lockdown. Before the lockdown, 30% were students, 54.7% were working, and 15.3% were not working, with the majority of working individuals being males (68%) (*p*-value < 0.001). During the lockdown, 27.3% were students, 45% were working, and 27.7% were not working. This shows that the proportion of working individuals of both males and females decreased during the lockdown, yet most of the employed individuals were males as well (58.5%) (*p*-value < 0.001). Although the majority of the study population has had no loss of income since lockdown (overall 62.4%, males and females being 57.5 and 67.3%, respectively, *p*-value < 0.001), most of them struggled to make money last till the end of the month (54.8%). Moreover, 71.2% of the study population had no struggle to have enough money for shopping, with males and females representing 70.1 and 72.3% of the overall proportion (*p*-value = 0.008).

**Table 1:** Sociodemographic and socioeconomic characteristics of the study population.

**Variables**	**Overall (12,447)**	**Male (6,213; 49.9)**	**Female (6,234; 50.1)**	***P*-values**
**Mean** **±SD**
Age	33.2 ± 12.9	34.9 ± 13.6	31.5 ±11.8	< 0.001
Adolescents	18.0 ± 0.0	18.0 ± 0.0	18.0 ± 0.0	< 0.001
Youth	21.0 ± 1.3	20.9 ± 1.3	21.0 ± 1.3	
Adult	38.5 ± 10.6	39.4 ± 11.0	37.59 ± 10.0	
Elderly	67.5 ± 4.3	67.4 ± 4.2	68.0 ± 4.6	
***N*** **(%)**
Age				< 0.001
Adolescents	490 (3.9)	260 (4.2)	230 (3.7)	
Youth	3,555 (28.6)	1,448 (23.3)	2,107 (33.8)	
Adult	8,180 (65.7)	4,335 (69.8)	3,844 (61.7)	
Elderly	223 (1.8)	170 (2.7)	52 (0.8)	
Gender				NA
Male	6,213 (49.9)	NA	NA	
Female	6,234 (50.1)			
Countries				< 0.001
Bahrain	1,208 (9.7)	577 (9.3)	631 (10.1)	
Egypt	1,331 (10.7)	741 (11.9)	590 (9.5)	
Jordan	1,290 (10.4)	683 (11.0)	607 (9.7)	
Kuwait	1,297 (10.4)	688 (11.1)	609 (9.8)	
Lebanon	1,229 (9.9)	601 (9.7)	627 (10.1)	
Oman	1,163 (9.3)	500 (8.0)	663 (10.6)	
Qatar	1,269 (10.2)	652 (10.5)	617 (9.9)	
Saudi Arabia	1,205 (9.7)	568 (9.1)	637 (10.2)	
United Arab Emirates	1210 (9.7)	579 (9.3)	632 (10.1)	
Palestine	1,245 (10.0)	625 (10.1)	620 (9.9)	
Education				< 0.001
Under a high school diploma	500 (4.0)	278 (4.5)	222 (3.6)	
High school diploma or equivalent	2,528 (20.3)	1,323 (21.3)	1,206 (19.3)	
Bachelor's degree	6,852 (55.0)	3,132 (50.4)	3,720 (59.7)	
Master's degree	1,683 (13.5)	880 (14.2)	803 (12.9)	
Doctorate	884 (7.1)	600 (9.7)	284 (4.6)	
Employment before the lockdown				< 0.001
Student	3,733 (30.0)	1,522 (24.5)	2,211 (35.5)	
Working	6,805 (54.7)	4,222 (68.0)	2,582 (41.4)	
Didn't working	1,910 (15.3)	469 (7.5)	1,441 (23.1)	
Employment during the lockdown				< 0.001
Student	3,396 (27.3)	1,352 (21.8)	2,044 (32.8)	
Working	5,597 (45.0)	3,635 (58.5)	1,962 (31.5)	
Didn't working	3,453 (27.7)	1,226 (19.7)	2,227 (35.7)	
Loss of income since lockdown				< 0.001
Yes	4,679 (37.6)	2,640 (42.5)	2,039 (32.7)	
No	7,768 (62.4)	3,573 (57.5)	4,195 (67.3)	
Struggle to make money last until the end of the month				0.528
No	5,631 (45.2)	2,829 (45.5)	2,803 (45.0)	
Yes	6,816 (54.8)	3,385 (54.5)	3,431 (55.0)	
Struggle to have enough money to shopping				0.008
No	8,864 (71.2)	4,357 (70.1)	4,507 (72.3)	
Yes	3,538 (28.8)	1,856 (29.9)	1,727 (27.7)	

Overall body mass index (BMI) among study participants significantly increased before and during the COVID-19 pandemic (26.2 and 26.4 kg/m^2^, respectively; *p*-value < 0.001) (Table 2). There is a minor change in the proportion of individuals who were underweight and obese, yet the proportion of normal BMI significantly decreased from before to during the pandemic (42.3 and 40.1%, respectively), and significantly increased for the overweight category from before to during the pandemic (31 and 33%, respectively; *p*-value < 0.001). A significant increase in BMI during the pandemic is also shown in both males and females (*p*-value < 0.001) (Table 2).

**Table 2:** Food groups consumption among study participants before and during the COVID-19 pandemic based on sex.

**Variables**		**Overall**			**Male**			**Female**			**Comparison**	
		**(12,447)**			**(6,213; 49.9)**			**(6,234; 50.1)**			**male-female**	
		**Before**	**During**	***P*-value**	**Before**	**During**	***P*-value**	**Before**	**During**	***P*-value**	***P*-value**	***P*-value**
											**Before**	**After**
BMI (Mean± SD)		26.3 ± 5.8	26.4 ± 5.8	< 0.001	26.9 ± 5.5	27.1 ± 5.4	< 0.001	25.6 ± 6.0	25.7 ± 6.0	< 0.001	< 0.001	< 0.001
Underweight *N* (%)		487 (4.1)	495 (4.2)	< 0.001	186 (3.1)	179 (3.0)	< 0.001	301 (5.1)	316 (5.3)	0.001	< 0.001	< 0.001
Normal *N* (%)		5,044 (42.3)	4,784 (40.1)		2144 (35.9)	1,982 (33.2)		2,901 (48.7)	2,802 (47.0)			
Overweight *N* (%)		3,695 (31.0)	3,936 (33.0)		2,149 (36.0)	2,318 (38.8)		1,545 (25.9)	1,619 (27.2)			
Obese *N* (%)		2,698 (22.6)	2,709 (22.7)		1,488 (25.0)	1,488 (25.0)		1,210 (20.3)	1,221 (20.5)			
**Food groups consumed** ***N*** **(%)**	**Frequency per week**	
Fruit (fresh or frozen) *N* (%)	4 or less	7,645 (61.4)	7,484 (60.1)	0.001	4,157 (66.9)	4,026 (64.8)	< 0.001	3,489 (56.0)	3,457 (55.5)	0.412	< 0.001	< 0.001
	5 or more	4,802 (38.6)	4,963 (39.9)		2,056 (33.1)	2,187 (35.2)		2,745 (44.0)	2,776 (44.5)			
Vegetables (fresh or frozen) N (%)	4 or less	5,810 (46.7)	6,256 (50.3)	< 0.001	3,328 (53.6)	3,547 (57.1)	< 0.001	2,482 (39.8)	2,710 (43.5)	< 0.001	< 0.001	< 0.001
	5 or more	6,637 (53.3)	6,191 (49.7)		2,885 (46.4)	2,666 (42.9)		3,752 (60.2)	3,524 (56.5)			
Legumes/pulses (e.g., beans, lentils, chickpeas) *N* (%)	4 or less	10,286 (82.6)	10,026 (80.5)	< 0.001	5,108 (82.2)	5,043 (81.2)	0.049	5,178 (83.1)	4,982 (79.9)	< 0.001	0.213	0.081
	5 or more	2,161 (17.4)	2,161 (19.5)		1,105 (17.8)	1,170 (18.8)		1,056 (16.9)	1,251 (20.1)			
Nuts *N* (%)	4 or less	9,740 (78.3)	9,722 (78.1)	0.709	4,919 (79.2)	4,950 (79.7)	0.348	4,821 (77.3)	4,772 (76.5)	0.139	0.013	< 0.001
	5 or more	2,707 (21.7)	2,725 (21.9)		1,294 (20.8)	1,263 (20.3)		1,413 (22.7)	1,462 (23.5)			
Processed meat/ poultry/fish/ vegetarian alternatives *N* (%)	4 or less	9,571 (76.9)	10,248 (82.3)	< 0.001	4,839 (77.9)	5,062 (81.5)	< 0.001	4,731 (75.9)	5,186 (83.2)	< 0.001	0.009	0.012
	5 or more	2,876 (23.1)	2,199 (17.7)		1,374 (22.1)	1,151 (18.5)		1,502 (24.1)	1,048 (16.8)			
Unprocessed fish	4 or less	11,156 (89.6)	10,814 (86.9)	< 0.001	5,543 (89.2)	5,354 (86.2)	< 0.001	5,613 (90.0)	5,460 (87.6)	< 0.001	0.132	0.02
	5 or more	1,291 (10.4)	1,633 (13.1)		670 (10.8)	859 (13.8)		621 (10.0)	774 (12.4)			
Unprocessed poultry *N* (%)	4 or less	9,701 (77.9)	9,411 (75.6)	< 0.001	4,996 (80.4)	4,772 (76.8)	< 0.001	4,705 (75.5)	4,638 (74.4)	0.049	< 0.001	0.002
	5 or more	2,746 (22.1)	3,036 (24.4)		1,217 (19.6)	1,441 (23.2)		1,529 (24.5)	1,595 (25.6)			
Unprocessed red meat *N* (%)	4 or less	10,734 (86.2)	10,487 (84.3)	< 0.001	5,308 (85.4)	5,219 (84.0)	0.002	5,426 (87.0)	5,267 (84.5)	< 0.001	0.009	0.443
	5 or more	1,713 (13.8)	1,960 (15.7)		905 (14.6)	994 (16.0)		808 (13.0)	966 (15.5)			
Whole wheat bread, pasta, grains *N* (%)	4 or less	9,200 (73.9)	8,876 (71.3)	< 0.001	4,749 (76.4)	4,594 (73.9)	< 0.001	4,450 (71.4)	4,282 (68.7)	< 0.001	< 0.001	< 0.001
	5 or more	3,247 (26.1)	3,571 (28.7)		1,464 (23.6)	1,619 (26.1)		1,783 (28.6)	1,952 (31.3)			
Milk *N* (%)	4 or less	7,543 (60.6)	7,391 (59.4)	0.001	4,039 (65.0)	3,927 (63.2)	0.001	3,504 (56.2)	3,464 (55.6)	0.217	< 0.001	< 0.001
	5 or more	4,904 (39.4)	5,056 (40.6)		2,174 (35.0)	2,286 (36.8)		2,730 (43.8)	2,770 (44.4)			
Other dairy products *N* (%)	4 or less	6,194 (49.8)	6,253 (50.2)	0.248	3,512 (56.5)	3,561 (57.3)	0.176	2,683 (43.0)	2,692 (43.2)	0.816	< 0.001	< 0.001
	5 or more	6,253 (50.2)	6,194 (49.8)		2,702 (43.5)	2,653 (42.7)		3,551 (57.0)	3,542 (56.8)			
Sweet snacks *N* (%)	4 or less	9,174 (73.7)	8,858 (71.2)	< 0.001	4,887 (78.7)	4,723 (76.0)	< 0.001	4,287 (68.8)	4,135 (66.3)	< 0.001	< 0.001	< 0.001
	5 or more	3,273 (26.3)	3,589 (28.8)		1,326 (21.3)	1,490 (24.0)		1,947 (31.2)	2,098 (33.7)			
Sugared beverages *N* (%)	4 or less	7,330 (58.9)	7,428 (59.7)	0.046	3,674 (59.1)	3,760 (60.5)	0.013	3,656 (58.7)	3,668 (58.8)	0.749	0.595	0.056
	5 or more	5,117 (41.1)	5,019 (40.3)		2,540 (40.9)	2,453 (39.5)		2,577 (41.3)	2,566 (41.2)			
Fats and oils *N* (%)	4 or less	9,825 (78.9)	9,716 (78.1)	0.012	4,883 (78.6)	4,843 (78.0)	0.206	4,942 (79.3)	4,873 (78.2)	0.023	0.342	0.768
	5 or more	2,622 (21.1)	2,730 (21.9)		1,330 (21.4)	1,370 (22.0)		1,291 (20.7)	1,361 (21.8)			
Food Consumption Score (Mean ± SD)		101.5 ± 46.6	101.8 ± 50.1	0.279	97.3 ± 47.7	98.3 ± 51.2	0.015	105.6 ± 45.2	105.2 ± 48.7	0.382	< 0.001	< 0.001
Food Consumption Score (*N*%)	Low	1,190 (9.6)	1,335 (10.7)	< 0.001	757 (12.2)	785 (12.6)	0.259	432 (6.9)	550 (8.8)	< 0.001	< 0.001	< 0.001
	High	11,257 (90.4)	11,112 (89.3)		5,456 (87.8)	5,429 (87.4)		5,802 (93.1)	5,683 (91.2)			
Percentage of decline in the Food Consumption Score		1.1			0.4			1.9			0.000	

### Food groups consumption patterns

#### Fruit and vegetable group

There was a significant change in food consumption patterns during the pandemic among males and females. The overall consumption of fruits increased (*p*-value < 0.001), though it was only significant in males (*p*-value < 0.001). Vegetable consumption decreased during the pandemic, in which the overall consumption of vegetables, in a frequency of 5 times per week, decreased from 53.3 to 49.7% from before to during the pandemic, respectively, and this decrease is shown in both genders (*p*-value < 0.001). Food consumption patterns were also analyzed among study participants before and during the pandemic based on BMI (Table 3). The consumption of fruits and vegetables was significantly higher in overweight participants before the lockdown compared to participants with normal BMI (*p*-value = 0.011 and 0.006, respectively). However, there was a significant increase in the consumption of fruits only during the pandemic in both subgroups (*P*-value < 0.001), while vegetable consumption of 5 or more per week significantly decreased in participants with normal BMI (24 and 22%, respectively, *p*-value = 0.002).

**Table 3:** Food groups consumption and food consumption scores among study participants before and during the COVID-19 pandemic based on self-reported BMI.

**Food groups consumed**	**Frequency per week**	**Before lockdown** ***n*** **(%)**			**During lockdown** ***n*** **(%)**			***P*** **-value**	
		**BMI**		***P*-value**	**Self-reported BMI**		***P*-value**	**Comparison before during**	
		**Normal**	**Overweight**		**Normal**	**Overweight**		**Normal**	**Overweight**
		**5,533 (46.4)**	**6,391 (53.6)**		**5,285 (44.3)**	**6,639 (55.7)**			
Fruit (fresh or frozen)	4 or less	3,448 (28.9)	3,837 (32.2)	0.011	3,177 (26.6)	3,949 (33.1)	0.485	< 0.001	< 0.001
	5 or more	2,085 (17.5)	2,554 (21.4)		2,108 (17.7)	2,690 (22.6)			
Vegetables (fresh or frozen)	4 or less	2,656 (22.3)	2,908 (24.4)	0.006	2,645 (22.2)	3,340 (28)	0.777	0.002	0.083
	5 or more	2,877 (24.1)	3,483 (29.2)		2,640 (22.1)	3,299 (27.7)			
Legumes/pulses (e.g., beans, lentils, chickpeas)	4 or less	4,584 (38.4)	5,267 (44.2)	0.531	4,177 (35)	5,426 (45.5)	< 0.001	< 0.001	< 0.001
	5 or more	949 (8)	1,124 (9.4)		1,108 (9.3)	1,213 (10.2)			
Nuts	4 or less	4,375 (36.7)	4,918 (41.2)	0.005	4,071 (34.1)	5,221 (43.8)	0.035	< 0.001	< 0.001
	5 or more	1,158 (9.7)	1,473 (12.4)		1,214 (10.2)	1,418 (11.9)			
Processed meat/poultry/fish/vegetarian alternatives	4 or less	4,226 (35.4)	4,932 (41.4)	0.306	4,271 (35.8)	5,553 (46.6)	< 0.001	< 0.001	< 0.001
	5 or more	1,307 (11)	1,459 (12.2)		1,014 (8.5)	1,086 (9.1)			
Unprocessed fish	4 or less	4,975 (41.7)	5,730 (48.1)	0.643	4,560 (38.2)	5,816 (48.8)	0.033	< 0.001	< 0.001
	5 or more	558 (4.7)	661 (5.5)		725 (6.1)	823 (6.9)			
Unprocessed poultry	4 or less	4,293 (36)	5,005 (42)	0.341	3,967 (33.3)	5,049 (42.3)	0.211	< 0.001	< 0.001
	5 or more	1,240 (10.4)	1,386 (11.6)		1,318 (11.1)	1,590 (13.3)			
Unprocessed red meat	4 or less	4,755 (39.9)	5,529 (46.4)	0.365	4,361 (36.6)	5,679 (47.6)	< 0.001	< 0.001	< 0.001
	5 or more	778 (6.5)	862 (7.2)		924 (7.7)	960 (8)			
Whole wheat bread, pasta, grains	4 or less	4,015 (33.7)	4,782 (40.1)	0.005	3,691 (31)	4,820 (40.4)	0.001	< 0.001	< 0.001
	5 or more	1,518 (12.7)	1,609 (13.5)		1,594 (13.4)	1,819 (15.3)			
Milk	4 or less	3,481 (29.2)	3,732 (31.3)	< 0.001	3,237 (27.1)	3,814 (32)	< 0.001	< 0.001	< 0.001
	5 or more	2,052 (17.2)	2,659 (22.3)		2,048 (17.2)	2,825 (23.7)			
Other dairy products	4 or less	2,880 (24.2)	3,001 (25.2)	< 0.001	2,717 (22.8)	3,235 (27.1)	0.004	0.383	0.057
	5 or more	2,653 (22.2)	3,390 (28.4)		2,568 (21.5)	3,404 (28.5)			
Sweet snacks	4 or less	3,949 (33.1)	4,858 (40.7)	< 0.001	3,648 (30.6)	4,834 (40.5)	< 0.001	< 0.001	< 0.001
	5 or more	1,584 (13.3)	1,533 (12.9)		1,637 (13.7)	1,805 (15.1)			
Sugared beverages	4 or less	3,259 (27.3)	3,768 (31.6)	0.95	3,153 (26.4)	3,993 (33.5)	0.591	< 0.001	< 0.001
	5 or more	2,274 (19.1)	2,623 (22)		2,132 (17.9)	2,646 (22.2)			
Fats and oils	4 or less	4,368 (36.6)	5,047 (42.3)	0.972	4,067 (34.1)	5,227 (43.8)	0.02	< 0.001	< 0.001
	5 or more	1,165 (9.8)	1,344 (11.3)		1,218 (10.2)	1,412 (11.8)			
Food Consumption Score	Low	623 (11.3%)	521 (8.2%)	< 0.001	612 (11.6%)	666 (10.0%)	< 0.001	< 0.001	< 0.001
	High	4,910 (88.7%)	5,870 (91.8%)		4,673 (88.4%)	5,973 (90.0%)		< 0.001	< 0.001
Percentage of decline in the Food Consumption Score		−3.1			−1.6		(*totaldecline*:1.5)	0.000	

#### Legumes/pulses, nuts and starchy food groups

There was an overall increase in the consumption of legumes and pulses during the pandemic in both genders. Males and females were significantly (*p*-value < 0.001) consuming more whole wheat, bread pasta and grains during (28.7%) in the pandemic as compared to the consumption before (26.1%) of this pandemic (Table 2). Food consumption patterns that were analyzed based on BMI show that legume/pulses consumption increased during the lockdown in both normal and overweight participants (9 and 10%, respectively, *p*-value < 0.001) (Table 3). Likewise, nuts consumption of 5 or more per week was also higher among overweight participants before the lockdown (*p*-value = 0.005), yet, it increased in normal participants, but decreased among overweight participants during the lockdown (*p*-value < 0.001). Overall, the consumption of starchy food (whole wheat, bread, pasta, and grains) was higher among overweight participants before the lockdown and increased during the pandemic in both normal and overweight participants (*p*-value < 0.001) (Table 3).

#### Protein group

Although consumption of processed meat, poultry, fish, and vegetarian alternatives significantly decreased during the pandemic in both sexes, the consumption of unprocessed fish, poultry and red meat significantly increased in both males and females with a *p*-value < 0.001 (Table 2). Moreover, food consumption patterns based on normal weight and overweight participants (Table 3), show that the consumption of processed meat, poultry, fish, and vegetarian alternatives decreased in both normal and overweight participants during the lockdown (8.5 and 9%, respectively, *p*-value < 0.001). However, the consumption of unprocessed fish, poultry, and red meat increased significantly in both subgroups during the lockdown (*p*-value < 0.001) (Table 3).

#### Milk and dairy

The milk consumption at a frequency of 5 or more per week before and during the pandemic was significantly different in both males and females (Table 2), but it differed obviously in men (*p*-value < 0.001) compared to women. Based on categories of BMI, the overall consumption of milk and other dairy products was higher among overweight participants before the lockdown, and significantly increased during the pandemic, while there was a significant decrease in normal weight subjects (*p*-value < 0.001) (Table 3).

#### Sweet snacks and sugared beverages

There was a significant increase in the consumption of sweet snacks and sugar-sweetened beverages in a frequency of 5 or more times per week before and during the pandemic (26.3 and 28.8% in males and females, respectively, *p*-value < 0.001). However, the consumption of sugared beverages significantly decreased in males (*p*-value = 0.013), but not in females (Table 2). Analyses of the consumption of sweet snacks based on BMI show that normal-weight participants had higher consumption of 5 or more times per week before the pandemic, yet, the increase in the consumption of sweets in overweight participants during the pandemic exceeded that in the normal BMI category (15 vs. 13.7%, respectively, *p*-value < 0.001) (Table 3). Other than that, there was a significant difference in the consumption of sugared beverages from before to during the lockdown in both normal and overweight BMI categories, in which the consumption of 4 or more per week decreased in normal BMI participants but increased in overweight participants (26.4 and 33.5%, respectively, *p*-value < 0.001) (Table 3).

#### Fats and oils

The overall consumption of fats and oils significantly increased during the pandemic (*p*-value = 0.012) as well as in females (*p*-value = 0.023), but not in males. Moreover, the analyzed consumption patterns among study participants before and during the pandemic based on BMI show that the proportion of participants who consumed fats and oils 5 times per week or more increased significantly among both subgroups of BMI during the pandemic (10.2 and 11.8%, respectively, *p*-value < 0.001) (Table 3).

### Food consumption score: An indicator of dietary diversity

The food consumption patterns of participants were compared between both periods (before and during the COVID-19 pandemic). The mean FCS ± SD before the pandemic was 101.5 ± 46.6, which was observed to be just the same during the pandemic period (101.8 ± 50.1), *p* = 0.279. However, when participants were stratified by their gender, the FCS appeared to increase significantly only among males from before the pandemic period (97.3 ± 47.7) to during the pandemic (98.3 ± 51.2), *p*-value = 0.015. The proportion of participants having low FCS (< 42) or an undiversified diet increased significantly from 9.6% (before the pandemic) to 10.7% during the pandemic period (*p*-value < 0.001). According to gender, the proportion of female participants with undiversified diet increased from 6.9 to 8.8% during the lockdown (*p*-value ≤ 0.001), while it (12.2–12.6%) remained just the same among males (*p*-value = 0.259) (Table 2).

Data based on BMI analysis showed that before the pandemic period, more normal-weight participants (11.3%) had an undiversified diet than overweight participants (8.2%), *p* < 0.001. These findings were also alike during the pandemic period, in which normal-weight participants with an undiversified diet exceeded that observed for overweight participants (11.6 vs. 10.0%, *p* < 0.001) (Table 3). Along with these findings, the proportion of normal-weight participants with an undiversified diet increased during the pandemic period (before: 11.3%; during: 11.6%, *p* < 0.001). Likewise, the proportion of overweight participants with a low diversity diet was 8.2% before the pandemic, reaching 10% during the pandemic period (*p* < 0.001). Taking into consideration both participants' gender and their BMI, normal-weight females and males had more low diversity in their diet (8.0 and 15.8%, respectively) than overweight females and males (5.7 and 10.0%, respectively) (*p* = 0.001 and *p* < 0.001, respectively), before the pandemic period. Similarly, during the pandemic period, normal-weight females and males had low diversity in their food groups intake (9.6 and 14.5%, respectively) than overweight females and males (7.6 and 11.9%, respectively) (*p* = 0.007 and *p* = 0.003, respectively) (data not shown).

### Determinants of self-reported-weight change

Table 4 shows the study variables determining participants' weight change toward higher BMI before and during the COVID-19 pandemic. These determinants were: the country of origin, gender, age, education level, working status, food-related behaviors, and the FCS (before the pandemic only).

**Table 4:** Backwards odds ratios (OR) according to body mass index (BMI).

	**Dependent variable (BMI**<**25 vs. BMI** > **25)**					
**Independent variable**	**Before lockdown**			**During lockdown**		
	**Odds ratio**	**OR confidence interval**	***P*-value**	**Odds ratio**	**OR confidence interval**	***P*-value**
Region: MENA vs. GULF	0.799	(0.738–0.866)	0.001	0.9	(0.831–0.975)	0.001
Gender: female vs. male	0.569	(0.524–0.618)	0.001	0.559	(0.515–0.607)	0.001
Age group: adolescents vs. youth	0.281	(0.187–0.424)	0.001	0.273	(0.183–0.406)	0.001
Age group: Adolescents vs. adults	0.394	(0.277–0.559)	0.001	0.387	(0.276–0.542)	0.001
Age group: adolescents vs. elderly	0.71	(0.511–0.986)	0.041	0.945	(0.687–1.301)	0.73
Education level: under a high school diploma vs. a high school diploma	0.642	(0.494–0.833)	0.001	0.641	(0.492–0.835)	0.001
Education level: Under a high school diploma vs. Bachelor's degree	0.637	(0.523–0.776)	0.001	0.693	(0.568–0.845)	0.001
Education level: under a high school diploma vs. master's degree	0.598	(0.501–0.713)	0.001	0.63	(0.527–0.754)	0.001
Education level: under a high school diploma vs. doctorate	0.491	(0.405–0.596)	0.001	0.512	(0.421–0.622)	0.001
Employment status: I was a student vs. I worked	0.64	(0.549–0.748)	0.001	0.717	(0.626–0.821)	0.001
Employment status: I worked vs. I didn't work	1.154	(1.027–1.296)	0.016	1.144	(1.036–1.263)	0.008
Plan meals to include all food groups: negative vs. positive	0.696	(0.629–0.770)	0.001	0.745	(0.662–0.838)	0.001
Think about healthy choices when deciding what to eat: positive vs. negative	1.051	(0.938–1.177)	0.392	1.028	(0.905–1.168)	0.668
Feel confident about managing money to buy healthy food: negative vs. positive	0.898	(0.810–0.995)	0.039	0.963	(0.860–1.078)	0.514
Use the nutritional information panel: positive vs. negative	1.022	(0.918–1.137)	0.696	1.067	(0.943–1.207)	0.305
Use other parts of the food label to make food choices: positive vs. negative	1.158	(1.041–1.289)	0.007	1.149	(1.014–1.302)	0.029
Cook meals at home using healthy ingredients: negative vs. positive	0.844	(0.744–0.958)	0.008	0.932	(0.810–1.073)	0.328
Feel confident about cooking a variety of healthy meals: positive vs. negative	1.093	(0.960–1.244)	0.18	1.079	(0.936–1.243)	0.296
Change recipes to make them healthier: negative vs. positive	0.85	(0.764–0.946)	0.003	0.867	(0.771–0.975)	0.017
Cook with leftover food: negative vs. positive	0.955	(0.877–1.039)	0.282	0.902	(0.826–0.984)	0.021
Throw away leftover food: negative vs. positive	1.082	(0.998–1.173)	0.055	1.089	(1.003–1.182)	0.043
FCS before lockdown: non-acceptable vs. acceptable	0.742	(0.650–0.848)	0.001	0.887	(0.781–1.007)	0.064

Participants from GULF countries were 20% (before the pandemic) and 10% (during the pandemic) more likely of being overweight compared to those from MENA counties (OR = 0.80, CI = 0.74–0.87, *p* = 0.001, and OR = 0.90, CI = 0.83–0.97, *p* = 0.001, respectively). Besides, males had about 44% more possibility to be overweight as opposed to females before and during the pandemic period (OR = 0.57, CI = 0.52–0.62, *p* = 0.001; OR = 0.56, CI = 0.51–0.61, *p* = 0.001). Youth, and adult participants had around 72 and 61% higher likelihood of being overweight compared to adolescents, respectively, before (OR = 0.28, CI = 0.19–0.42, *p* = 0.001; OR = 0.39, CI = 0.28–0.56, *p* = 0.001, respectively) and during the pandemic (OR = 0.27, CI = 0.18–0.40, *p* = 0.001 and OR = 0.39, CI = 0.28–0.54, *p* = 0.001, respectively). Elderly participants were 29% more likely to be overweight than adolescents before the pandemic period (OR = 0.71, CI = 0.511–0.986, *p* = 0.04). Regarding the education level, participants holding a high school diploma (before pandemic: OR = 0.64, CI = 0.49–0.83, *p* = 0.001; during pandemic: OR = 0.64, CI = 0.49–0.83, *p* = 0.001), Bachelor's degree (before pandemic: OR = 0.64, CI = 0.52–0.78, *p* = 0.001; after pandemic: OR = 0.69, CI = 0.56–0.84, *p* = 0.001), Master's degree (before pandemic: OR = 0.60, CI = 0.50–0.71, *p* = 0.001; during pandemic: OR = 0.63, CI = 0.53–0.75, *p* = 0.001) and Doctorate degree (OR = 0.49, CI = 0.41–0.60, *p* = 0.001; OR = 0.51, CI = 0.42–0.62, *p* = 0.001) were more vulnerable of being overweight, in contrast to other participants with high school diploma education level. In addition, workers had a 36 and 20% higher probability of being overweight compared to those who were still students (OR = 0.64, CI = 0.55–0.75, *p* = 0.001) or not working (OR = 1.20, CI = 1.02–1.30, *p* = 0.016) before the pandemic. This finding was also similar during the pandemic; workers had a 28 and 14% higher probability of being overweight (vs. students, OR = 0.72, CI = 0.63–0.82, *p* = 0.001; vs. non-workers, OR = 1.14, CI = 1.04–1.26, *p* = 0.008).

Furthermore, participants who used to plan meals to include all food groups were 30% (before the pandemic) and 26% (during the pandemic) more probable of being overweight compared to those who admitted not doing so (OR = 0.70, CI = 0.63–0.77, *p* = 0.001, and OR = 0.74, CI = 0.66–0.84, *p* = 0.001, respectively). Besides, participants who reported using other parts (like ingredients) of the food label to make food choices had around a 16% higher probability to be overweight compared to others who did not use this (before pandemic: OR = 1.16, CI = 1.04–1.29, *p* = 0.007; during a pandemic: OR = 1.15, CI = 1.01–1.30, *p* = 0.029). Those who reported feeling confident about managing money to buy healthy food were 10% more likely of suffering from overweight (OR = 0.90, CI = 0.81–0.99, *p* = 0.039) compared to their counterparts before the pandemic. Those who used to cook meals at home using healthy ingredients had a 16% more likelihood to be overweight compared to their counterparts (OR = 0.84, CI = 0.74–0.96, *p* = 0.008) before the pandemic. In addition, participants who claimed to change recipes to make them healthier were 15% (before pandemic: OR = 0.85, CI = 0.76–0.95, *p* = 0.003) and 13% (during pandemic: OR = 0.87, CI = 0.77–0.97, *p* = 0.017) more likely to be overweight (OR = 0.85, CI = 0.76–0.95, *p* = 0.003). Participants with an acceptable FCS appeared to have 26% more vulnerability to being overweight as opposed to other participants classified as having low FCS before the pandemic (OR = 0.74, CI = 0.650–0.848, *p* < 0.001). Lastly, those who reported cooking with and throwing away leftover foods had 10% and just 1% more susceptibility to being overweight during the pandemic (OR = 0.90, CI = 0.83–0.98, *p* = 0.021, and OR = 1.01, CI = 1.003–1.182, *p* = 0.043, respectively).

## Discussion

This study showed that, during the COVID-19 period, the dietary diversity, declined by 1.9% among females compared to males (0.4%) (*p* < 0.001). During the pandemic, the consumption of the following food groups has increased: fruits, legumes/pulses, unprocessed (fish, poultry, and red meat), starchy foods (whole wheat, bread, pasta, and grains), milk, sweet snacks, and fats and oils. However, the consumption of vegetables (fresh or frozen), non-milk dairy products, processed (meat, poultry, fish, vegetarian alternatives), and sugared beverages had decreased. Besides, the FCS of overweight participants was significantly higher before and during the pandemic than their counterparts. Participants' country of origin (MENA vs. GULF), gender, age, education, working status, the FCS, and multiple food-related behaviors had predicted self-reported body weight change during the COVID-19 pandemic.

Study findings showed that the proportion of working individuals decreased during the lockdown, with higher job losses among females. After the emergence of the COVID-19 pandemic, the unemployment rate soared to levels not seen since the 1930s (16). Tens of millions of people lost their employment in the early months of the lockdown (16). Furthermore, according to a recent study, women are more affected by job losses in times of economic instability than men (17), and the COVID-19 pandemic was more severe for women than for males (18). Furthermore, according to Danielsen et al., it is predicted the gender gap to widen, pushing millions of women into poverty as a result of the COVID-19 pandemic (19, 20).

In terms of weight gain, the present study revealed that there was a significant increase (*p* < 0.001) in BMI among study participants during the COVID-19 confinement period. This result is in concordance with a longitudinal study conducted in Saudi Arabia by Alshahrani et al. (21), which documented a significant weight gain of 0.33 kg. In addition, 4.8% of normal-weight participants became overweight or obese, and about 5.1% of overweight participants suffered from obesity. In the same manner, El Zoghbi et al. (22) reported that the number of overweight and obese students increased by 5.2% in a study conducted on 174 Lebanese students before and at the end of the COVID-19 lockdown. Consistent findings were also reported in Srilanka (23), Brazil (24), Spain (25), and Bangladesh (26) where participants gained weight during the pandemic period. These findings are distressing because increasing adiposity had been associated with higher odds for COVID-19-related mortality in previous trials. For every unit increase in BMI, WHR (waist-to-hip ratio), and body fat, the odds of death amongst COVID-19 infection among participants increased by 1.04 (95% CI 1.01–1.07), 10.71 (95% CI 1.57–73.06) and 1.03 (95% CI 1.01–1.05), respectively (all *p* < 0.05) (27). Besides, our study results indicated that the BMI was significantly higher among males than females. This result was consistent with that of a recent study conducted by Nasui et al. (28) among Information Technology staff from Romania. Similarly, a study in Italy showed that the observed weight increase during the lockdown period was higher in male than in female adolescents (3.8 ± 3.4 vs. 1.2 ± 3.7 kg, *p* = 0.02) (29).

During the pandemic, the consumption of fruits, legumes/pulses, unprocessed (fish, poultry, and red meat), starchy foods (whole wheat, bread, pasta, and grains), milk, sweet snacks, and fats and oils increased in the overall population. However, the consumption of vegetables (fresh or frozen), non-milk dairy products, processed (meat, poultry, fish, vegetarian alternatives), and sugared beverages declined. This emerged pattern of food consumption during the lockdown was approximately similar to a study conducted by Mazzolani et al. (6), which reported that during the pandemic, there was a higher frequency of cooking, use of delivery service, and a higher prevalence of snacking. Also, consistent partially with our study findings, a systematic review provided information about a shift toward modified eating behaviors during the pandemic period, manifested by frequent snacking, and a preference for sweets and ultra-processed food (30). Besides, an experience in Lebanon showed that, during the COVID-19 pandemic, 44.7% of participants did not eat fruits daily, and 35.3% did not eat vegetables on daily basis, which confirms some of our findings (31). The impact of the lockdown period on diet and eating habits is wide and multi-sectoral. Although it has a direct influence on the daily amount of calories consumed, it alters also the diet composition, the driving factors to eating, between meals eating patterns and many other diets and lifestyle-related factors. These latter changes collectively shape the change in body weight, as observed globally since the confinement period starts.

In terms of the food consumption score, the proportion of participants having low FCS (< 42) increased significantly from 9.6% (before the pandemic) to 10.7% during the pandemic period (*p*-value < 0.001). This is consistent with a recent study in Lebanon showing that the FCS decreased by 4.6% among 2,822 Lebanese participants; however, the decrease was observed in the consumption of fruits (5.4% decrease, *p*-value < 0.001), vegetables (6.9% decrease, *p*-value < 0.001), processed meats, poultry, and fish (5.8% decrease, *p*-value < 0.001), other dairy products (5.1% decrease, *p*-value < 0.001), sweet snacks (3.1% decrease, *p*-value = 0.001), sugared beverages (3.4% decrease, *p*-value < 0.001), fats and oils (2.8%, *p*-value = 0.001), respectively (14). According to gender, the proportion of female participants having low FCS increased from 6.9 to 8.8% during the lockdown (*p*-value ≤ 0.001), while it remained just the same among males (*p*-value = 0.259). A study conducted by Shahbaz et al. (4) confirmed gender disparities regarding dietary diversity; male-headed households consumed more diversified food than female-headed households. The latter is also consistent with a study in Tanzania, showing that women and children access less diverse diets and achieve minimum dietary diversity (32). Data based on BMI analysis, the FSC of overweight participants was significantly higher than normal-weight participants before and during the pandemic period. This is explained by the fact that a higher dietary diversity leads probably to higher energy intake and leads to weight gain in some circumstances. These findings come in disagreement with the results of a study conducted by Chen et al. (33) among students in Selangor, Malaysia, which found that students with lower BMI (<23.0 kg/m^2^) had better food consumption patterns than those with higher BMI (≥23.0 kg/m^2^).

The regression findings showed that participants from GULF countries were more probably to be overweight than those from the MENA region not only before but also during the pandemic period. Many factors are responsible for the increased prevalence of overweight and obesity in the GULF region, including social and cultural environments, education, physical activity, diet and nutrition, and high income (34). Obesity in the Arabian Gulf is related to income growth due to the rich deposits of oil reserves, rapid urbanization, and improved living conditions (35). Upon this, the World Health Organization (WHO) reported that GULF countries have the highest rates of obesity, with the top ten countries including Kuwait, Bahrain, Saudi Arabia, and the United Arab Emirates^2^. Besides, in both study periods, males showed a higher probability of being overweight as opposed to females. Coming hand in hand with the latter finding, it was reported by WHO in 2016 that Japan, Korea, China, Germany, France, the United Kingdom, and the United States of America had a higher prevalence of obesity among men than females (36). Note that overweight problems by gender are much greater than obesity (36). We may relate these findings to the fact that males are usually less concerned about their weight status and lack nutrition knowledge. Regarding the age of the participants, adolescents showed a lower probability of having overweight compared to other age groups (youth, adults, and elderly) in both study periods. These findings are consistent with other reports showing that younger age is associated usually with a lower prevalence of obesity (37). Furthermore, in our study, a higher education level is associated with a higher risk of being overweight before and during the pandemic. One possible explanation for this finding is that higher education is usually coupled with higher income, especially in GULF countries, leading to adopting western lifestyles in their diets, causing weight gain in many circumstances. In addition, working participants were more probably to be overweight before and during the pandemic periods. This may be also related to the higher income, which leads mostly to adopt unhealthy diet-related behaviors, including fast food consumption and higher frequency of food consumption and snacking, for instance.

Also, a finding of the regression analysis is that participants who used to plan meals to include all food groups were more probable of being overweight compared to those who admitted to not doing so before and during the pandemic period. The consumption of more food groups may be associated probably with higher energy intake leading to weight gain over time. Besides, those who reported feeling confident about managing money to buy healthy food, using other parts (like ingredients) of the food label to make food choices, and those who claimed to change recipes to make them healthier, were more likely to suffer from overweight compared to their counterparts, in the pre-pandemic and during the pandemic period. The latter finding may be because healthy food choices may be also energy-dense, and weight gain is determined predominately by the amount of food eaten and the overall calories consumed. Packaged and prepared foods (even the healthy option, such as protein bars, granola, bread, and baked goods) can be calorie-dense and easy to overeat. Besides, before the pandemic, participants with an acceptable FCS appeared to be 26% more likely of being overweight as opposed to other participants classified as having low FCS. Dietary diversity has also been linked to overweight and abdominal obesity in preliminary investigations among Iranian children and adolescents (38). However, a meta-analysis failed to show a significant association between overweight/obesity comparing the highest and lowest diverse diets (39). The most rational explanation in our case is that a diversified diet contains more food ingredients which increase the caloric content of meals and lead to weight gain after some time.

## Strengths and limitations

The study included a few limitations which should be considered when evaluating the applicability of our findings. First, the cross-sectional design of the study limits reaching a causal inference. Second, recall bias is not unexpected, as the study included retrospective data to recall the food groups' consumption and patterns before the lockdown. Third, the questionnaire was self-administered and quite long, which increases the possibility of information bias. Despite these limitations, the value of the study is in its originality and novelty, being the first of its kind in our region. In addition, the large sample size in this study strengthens the applicability and generalizability of our findings.

## Conclusion

The current findings shedding light on the decline of dietary diversity and the increase in body weight in the Arab region are alarming and require immediate attention. These alarming findings call for emergency food security and dietary patterns policies to mitigate the burden of the health pandemic, along with other overlapping economic crises due to the pandemic, on both genders and to promote resilience for future shocks. Evidence-based and context-specific policies and strategies that can address the gender-responsive policies and recovery plans dimensions and pillars of healthy dietary patterns and food security are needed.

## Data availability statement

The raw data supporting the conclusions of this article will be made available by the authors, without undue reservation.

## Ethics statement

The studies involving human participants were reviewed and approved by University of Antwerp.

## CORONA COOKING survey regional study group

**Belgium**: Charlotte De Backer, Lauranna Teunissen, Kathleen Van Royen, Isabelle Cuykx, Paulien Decorte, Gaëlle Ouvrein, Karolien Poels, Heidi Vandebosch, Katrien Maldoy (University of Antwerp); Sara Pabian (Tilburg University), Christophe Matthys, Tim Smits, Jules Vrinten (KULeuven); Ann DeSmet (University of Antwerp, Université Libre de Bruxelles); Nelleke Teughels, Maggie Geuens, Iris Vermeir, Viktor Proesmans, Liselot Hudders (Ghent University);

**Bahrain**: Mariam Al-Mannai, Tariq Alalwan (University of Bahrain);

**Lebanon**: Elissa Naim (Lebanese University), Rania Mansour (Lebanese University), Nour Yazbeck (Lebanese University);

**Palestine**: Hazem Agha, Rania Abu Seir (Al Quds University);

**Saudi Arabia**: Jamila Arrish, Ghadir Fallata, Omar Alhumaidan, Shihana Alakeel, Norah AlBuayjan, Sarah Alkhunein, Budur Binobaydan, and Aeshah Alshaya (National Nutrition Committee (NNC) at Saudi Food and Drug Authority (Saudi FDA);

**United Arab Emirates**: Ayesha Aldhaheri (United Arab Emirates University).

## Author contributions

CB, LT, IC, PD, SP, and KV: conceptualization, software, validation, supervision, project administration, and funding acquisition. CB, LT, IC, PD, SP, KV, MH, HM, RM, MA, KB, RT, HAS, LC, RQ, RA, IK, SD, SA, MA-M, HB, and MW: methodology. RT, MI, HAS, HM, and MH: formal analysis, data curation, and writing—original draft preparation. RT, MI, HAH, and MH: investigation. CB, LT, IC, PD, SP, and KV resources. All authors and the research group contributed to the article and approved the submitted version.

## Funding

This research was funded by the Research Foundation Flanders (G047518N) and Flanders Innovation and Entrepreneurship (HBC.2018.0397). These funding sources had no role in the design of the study, the analysis and interpretation of the data or the writing, nor the decision to publish the manuscript.

## Conflict of interest

The authors declare that the research was conducted in the absence of any commercial or financial relationships that could be construed as a potential conflict of interest.

## Publisher's note

All claims expressed in this article are solely those of the authors and do not necessarily represent those of their affiliated organizations, or those of the publisher, the editors and the reviewers. Any product that may be evaluated in this article, or claim that may be made by its manufacturer, is not guaranteed or endorsed by the publisher.

## Footnotes

^1^https://www.uantwerpen.be/en/projects/food-media-society/corona-cooking-survey/; https://osf.io/8gpzx.

^2^https://apps.who.int/infobase/Comparisons.aspx (accessed June 22, 2022).
